# Soil quality enhancement drives tree growth and broadleaf dominance in fir-broadleaf mixed plantations

**DOI:** 10.3389/fpls.2025.1705626

**Published:** 2025-11-19

**Authors:** Zhigao Fu, Yihua Xiao, Shirong Liu, Han Xu, Yan Wang, Huosheng Zhu

**Affiliations:** 1 Research Institute of Tropical Forestry, Chinese Academy of Forestry, Guangzhou, China; 2 Pearl River Delta Farmland Shelterbelt Ecosystem Research Station, National Forestry and Grassland Administration, Guangzhou, China; 3 Key Laboratory of Forest Ecology and Environment of National Forestry and Grassland Administration, Ecology and Nature Conservation Institute, Chinese Academy of Forestry, Beijing, China; 4 Baotianman Forest Ecosystem Research Station, National Forestry and Grassland Administration, Nanyang, Henan, China; 5 Lechang Forest Farm of Guangdong Province, Forestry Administration of Guangdong Province, Lechang, Guangdong, China

**Keywords:** soil quality index, stand structure, soil properties, minimum data set, net primary productivity (NPP)

## Abstract

**Introduction:**

Evaluating soil quality is essential for guiding reforestation and land management strategies, particularly in degraded Chinese fir plantations where long-term productivity and successional dynamics remain poorly understood.

**Methods:**

This study assessed ten mixed-species planting patterns to quantify the Soil Quality Index (SQI) using a Minimum Data Set (MDS) approach, which reduces data redundancy by statistically identifying key indicators from a larger dataset, thereby effectively capturing essential soil functions, and subsequently explored the relationships between SQI and stand growth, structural diversity, biomass, net primary productivity (NPP), as well as percentage of broadleaf species (PBS).

**Results:**

Significant differences were observed across planting patterns in diameter at breast height (DBH), tree height (TH), stand biomass (FB), structural diversity (variation in DBH [CVD] and Gini coefficient [GiniD]), and PBS. Soil properties—including physical (soil moisture), chemical (soil organic carbon [SOC], total nitrogen [TN], total phosphorus [TP], ammonium nitrogen [NH₄⁺], nitrate nitrogen [NO₃⁻], available phosphorus [AP]), microbial (microbial biomass carbon [MBC], nitrogen [MBN], and phosphorus [MBP]), and enzymatic (e.g., peroxidase [POD], alkaline phosphatase [ALP], urease [URE])—also varied significantly. SQI values ranged from 0.42 to 0.65, with patterns Fir–Mytilaria laosensis mixed (ML), Fir–Castanopsis hystrix mixed (CH), Fir–Michelia chapensis mixed (MC), and Fir–Schima superba mixed (SS) associated with both high SQI and greater biomass. Sensitivity analysis identified Fir–Cinnamomum porrectum mixed (CP), ML, and SS as particularly responsive to hybridization. Among soil factors, URE, AP, and MBC were key drivers of productivity, while URE, AP, MBC, and POD significantly predicted the proportion of broadleaf trees. Enhanced soil quality was positively associated with increases in DBH, TH, and PBS, accelerating the successional transition from fir-dominated to broadleaf-dominated stands. However, SQI was not significantly correlated with structural diversity metrics.

**Discussion:**

These results underscore the importance of rational species selection in restoring degraded fir plantations and demonstrate that improving soil quality is a critical mechanism promoting near-natural forest succession.

## 1 Introduction

Soil is fundamental for plant growth and development (Li et al., 2020). Soil quality directly influences the biological productivity and functioning of forests (Liang et al., 2022). Furthermore, soil quality has been defined as "the ability of soil to sustain plant and animal productivity, enhance water and air quality, and support human health and habitation within natural ecosystems" (Li et al., 2019a). Consequently, evaluating soil quality is crucial for the advancement of sustainable forestry practices (Yu et al., 2023).

Since the release of the United States Department of Agriculture (USDA) classification system for land potential in 1961, numerous soil quality assessment methods have been developed (Atalay, 2016). The Soil Quality Index (SQI) is a widely utilized method for assessing soil function (Li et al., 2019b; Qian et al., 2023). Soil function generally can be evaluated through indicators of soil physicochemical and biochemical properties (Bünemann et al., 2018). However, the extensive range of physical, chemical, and biological properties complicates the measurement process, making it impractical to consider all these properties (Li et al., 2019b). Currently, the minimum data set (MDS), developed by Larson and Pierce, is arguably the most commonly employed method for evaluating SQI (Li et al., 2019a; Raiesi and Beheshti, 2022; Qian et al., 2023, Qian et al., 2023). This approach comprehensively accounts for the combined influence of measurements, weights, and interactions between indicators on the assessment results (Rezaei et al., 2006). Mixed forests markedly enhance soil quality by improving soil structure, augmenting nutrient availability, and increasing fungal diversity along with the stability of microbial networks (Ge et al., 2025). This advantage over pure coniferous forests is consistently observed across various ecosystems, with coniferous-broadleaf mixtures generally proving superior in maintaining and enhancing soil quality (Shao et al., 2020). This trend is also evident in karst-degraded regions, where mixed forests significantly increase soil organic carbon (SOC) and total nitrogen (TN) levels, thereby improving overall soil fertility and quality (Guan and Fan, 2020). Collectively, the evidence indicates that mixed forests achieve significantly higher Soil Quality Index (SQI) values compared to coniferous forests, highlighting the importance of mixed afforestation in sustaining soil quality.

Planted forests are recognized as an effective strategy for improving the quality of degraded soils and mitigating soil erosion throughout the world (Guo et al., 2021). *Chinese Fir*, as a major plantation species in southern China, is characterized by its straight stem shape, fast-growing and productive nature, strong adaptability, and ease of management and harvesting (Guo et al., 2022). *Chinese Fir* is extensively cultivated across 16 provinces in China (Li et al., 2024), with fir plantation forests comprising 24% of the total area of plantation forests in the country and 6% of plantation forests worldwide (Hemati et al., 2020). However, studies have reported that continuous cultivation and short-rotation harvesting associated with fir plantations are diminishing soil quality (Farooq, 2019). To date, extensive literature indicates that monoculture plantation forests encounter issues such as low biodiversity, as well as declining soil fertility and water-holding capacity (Chen et al., 2021; Li et al., 2021), which severely impede the advancement of modern sustainable forestry (Guo et al., 2023). Consequently, the establishment of mixed forests has become a central focus of various forest management strategies (Bauhus et al., 2017). The introduction of broadleaf trees into coniferous forests represents a crucial approach to enhancing carbon stocks within forest stands (Feng et al., 2022).

Incorporating broadleaf species into Fir plantations can improve soil physicochemical properties, accelerate soil nutrient turnover, and enhance stand productivity (Hou et al., 2021; Gao et al., 2022; Guo et al., 2023). Moreover, while the benefits of species mixing in enhancing forest structural stability and productivity are well documented (Hua et al., 2022), the impact on soil quality and fertility (as measured by the soil quality index) resulting from the conversion of cedar monoculture plantations to mixed forests remains largely unexplored in southern China (Guo et al., 2023). Therefore, there is an urgent need to evaluate soil quality, as maintaining or improving soil fertility is essential for sustainable forestry (Ye et al., 2022).

Forest stand modification through the introduction of different tree species is a common strategy in forestry production and management (Liu et al., 2016). Such modifications may lead to changes in vegetation diversity and shifts in ecological niche utilization, which can, in turn, affect forest structure and ecosystem stability (Guo et al., 2023). Stand structure plays a crucial role in predicting forest growth and productivity (Wang et al., 2021). The diversity of stand structure, characterized by variability in individual tree size, such as the Gini coefficient and the coefficient of variation in tree diameter (Ali, 2019) is increasingly recognized as a significant driver of ecosystem functioning (LaRue et al., 2019; Ouyang et al., 2023). Tree mortality is a fundamental process in forest succession (Zhang et al., 2020). Stand structure influences variations in competitive intensity and resource utilization, thereby affecting both tree mortality (Kweon and Comeau, 2019) and forest succession (Zhang et al., 2020). Current studies have raised concerns regarding the relationship between stand structure and productivity in mixed fir stands (Wang et al., 2021), the impacts of changes in stand density on stand structure (Chen et al., 2021; Liu et al., 2022; Sun et al., 2023), and the effects of stand structure and climatic factors on tree mortality (Zhang et al., 2020). However, the influence of changes in soil quality on stand structure and forest succession has been infrequently investigated. Exploring the relationship between soil quality, stand structure, and forest succession will not only provide a foundation for the scientific management of *Fir* plantation forests and the adjustment of local forestry policies, but will also serve as a reference for the sustainable development of the plantation forest industry globally.

Different tree species configurations were selected for stand rejuvenation at a fourth-generation *Fir* forest logging site in the southern subtropics. This paper employs the model for soil quality evaluation (MDS) to assess the differences in soil quality among various stand types. Additionally, it explores the relationships between soil quality and stand growth, biomass, structural diversity, and community succession to broadleaf species, expressed as a percentage of broadleaf species. The study hypothesizes that improved soil quality is positively associated with stand growth, biomass, and structural diversity, and soil quality improvement mediates tree size dynamics (DBH, TH) to accelerate competitive exclusion of fir by broadleaf species in mixed stands.

## 2 Materials and methods

### 2.1 Study area descriptions

**Figure 1:**
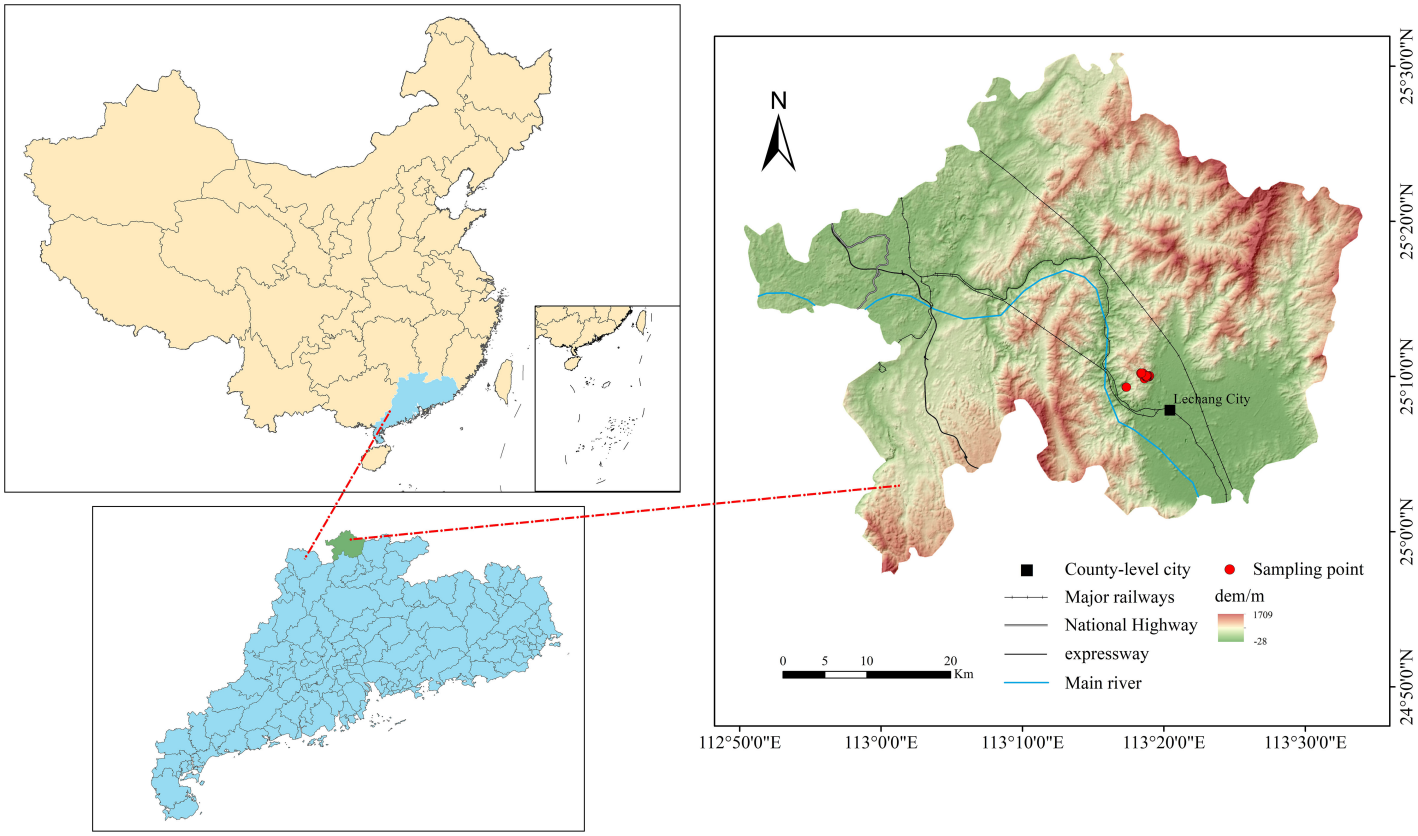
Map of the study area.

The study was conducted in Lechang Forest Farm, located in Guangdong Province, China (25°06'–25°19'N, 113°11'–113°23'E; **Figure 1**). The site spans 5,398.3 hectares and experiences a meso-subtropical monsoon climate, with a mean annual temperature of 19.6 °C, average annual precipitation of 1,522.3 mm, and mean annual relative humidity of 76%.

The *Fir* plantation forest in the study area has been cultivated for four generations, resulting in significant soil fertility decline and general forest degradation. The area was last logged in 2003, after which sprouting vegetation was retained. Following the logging event, the affected areas were replanted with a variety of broadleaf species, including *Mytilaria laosensis*, *Michelia macclurei*, *Michelia maudiae*, *Cinnamomum porrectum*, *Castanopsis hystrix*, *Cinnamomum burmanni*, *Liquidambar formosana*, *Michelia chapensis*, *Schima superba*, and *Michelia odora*, among others. In total, ten broadleaf and coniferous species were introduced to establish mixed stands, with broadleaf species deliberately interplanted among the remaining firs.

Land preparation involved band reclamation and the construction of weed-branch berms, while weeding and maintenance were conducted during the first three years after planting. This process led to the development of a mixed forest stand composed of both modified tree species and naturally regenerating fir trees, with an initial planting density of 3,975 trees per hectare.

In July 2023, representative stands of each of the ten tree species were selected, and three permanent plots (20 m × 30 m) were established for each species, with a minimum spacing of 20 m between plots. Within each plot, all trees were surveyed individually. Recorded data included species identity, diameter at breast height (DBH), tree height, height to first branch, spatial coordinates, and general growth condition. Further plot details are provided in **Table 1**.

**Table 1:** Basic characteristics of different tree species plantations for sample plots.

Abbreviations	Planting types	Altitude (m)	Aspect	Slope (°)	Density (Plant·hm^−2^)
ML	*Fir*–*Mytilaria laosensis*	448~465	southeast	23~28	800~917
MM	*Fir*–*Michelia macclurei*	378~466	southeast	18~22	2566~3066
MME	*Fir*–*Michelia maudiae*	452~461	southwest	16~18	2800~3450
CP	*Fir*–*Cinnamomum porrectum*	524~535	southeast	15~19	2700~2933
CH	*Fir*–*Castanopsis hystrix*	467~506	southwest	27~29	1033~1600
CB	*Fir*–*Cinnamomum burmanni*	409~449	northwest	13~20	675~1750
LF	*Fir*–*Liquidambar formosana*	367~466	southeast	21~26	1916~2375
MC	*Fir*–*Michelia chapensis*	415~420	southeast	16~19	1275~1775
SS	*Fir*–*Schima superba*	468~501	southeast	25~27	2000~3075
MO	*Fir*–*Michelia odora*	452~456	southwest	15~20	2175~2475

### 2.2 Soil sampling and soil properties analysis

Each 20 × 30 m sample plot was subdivided into six 10 × 10 m subplots. After removing surface litter, soil was sampled. Using a 5 cm diameter auger, nine surface soil cores (0–10 cm) were collected in an S-shaped pattern within each subplot. This sampling depth was selected as it represents the primary zone influenced by litter decomposition, root activity, and microbial processes, and it is the standard for soil quality assessments in subtropical plantations, where fine roots and nutrient cycling are predominantly concentrated. These cores were combined to form a composite sample. Additionally, three undisturbed soil samples were collected per subplot using a ring knife to determine bulk density and gravimetric moisture content. All soil samples were passed through a 2 mm sieve after removing visible roots and gravel. The sieved samples were stored at low temperature in a mobile refrigerator and transported to the laboratory, where each sample was divided into three portions: (1) air-dried for physicochemical analysis and amino sugar quantification; (2) refrigerated at –4 °C for biological analysis; and (3) retained as backup. Detailed analytical procedures for soil physicochemical and biological measurements are provided in **Appendix 1**.

### 2.3 Structural diversity and biomass of different mixed planting patterns

Structural diversity was quantified using variation in tree diameter at breast height (DBH), a standard metric for stand structure (Brejda et al., 2000; Schnabel et al., 2019). DBH variation was assessed by calculating the coefficient of variation (CV), defined as the standard deviation of DBH divided by the mean DBH (Wan et al., 2024). The Gini coefficient was also calculated to evaluate inequality in DBH distribution (Cordonnier and Kunstler, 2015).


$${\text{Gini}}_{\text{D}}=100\%\frac{\sqrt{\frac{1}{n}{\left(DBH_{i}-\text{DBH}\right)}^{2}}}{\text{DBH}}$$

where *Gini_D_*​ is the Gini coefficient, *n* is the number of trees in the plot, *DBH_i_​* is the DBH of the ith tree, and DBH is the mean DBH.

Due to large variation in tree size, species composition was quantified using biomass proportion rather than tree counts per hectare. Aboveground biomass was estimated using species-specific allometric equations incorporating DBH and tree height (Návar, 2009):


$$W=a{\left(D^{2}H\right)}^{b}$$

Where *W* is biomass (kg), *D* is DBH (cm), *H* is height (m), and *a* and *b* are species-specific coefficients (see **Appendix II**). Total plot biomass was calculated by summing individual tree biomass and scaled to per-hectare values. Net primary productivity was estimated as the average annual increment of aboveground biomass, defined as total biomass divided by stand age (Luo and Chen, 2011).

### 2.4 Soil quality assessment

Soil quality was assessed using the Soil Management Assessment Framework (SMAF), with the Soil Quality Index (SQI) calculated based on a Minimum Data Set (MDS) derived from a Total Data Set (TDS) of 20 soil indicators (Yu et al., 2023). Indicators were chosen to represent physical, chemical, and biological soil functions relevant to nutrient cycling, carbon dynamics, and structural stability (Nosrati, 2013; Guo et al., 2017; He et al., 2022). The TDS included soil moisture (SM), bulk density (BD), soil organic carbon (SOC), total nitrogen (TN), total and available phosphorus (TP, AP), ammonium (NH_4_^+^) and nitrate (NO_3_^-^) nitrogen, microbial biomass carbon (MBC), nitrogen (MBN), and phosphorus (MBP), and nine enzymes involved in C, N, and P cycling: peroxidase (POD), β-xylosidase (BX), cellobiohydrolase (CBH), β-glucosidase (BG), urease (URE), N-acetyl-β-D-glucosaminidase (NAG), leucine aminopeptidase (LAP), acid phosphatase (ACP), and alkaline phosphatase (ALP).

Kaiser–Meyer–Olkin (KMO = 0.704) and Bartlett’s test (*p* < 0.01) confirmed the suitability of the dataset for Principal Component Analysis (PCA). Indicators with loadings ≥ 0.5 on principal components (PCs) with eigenvalues ≥ 1 were grouped by PC and ranked using a normalized contribution metric:


$$N_{ik}=\sqrt{\sum_{i=1}^{k}(U_{ik}^{2}\cdot e_{k})}$$

Where *N_ik_* is the normalized loading of indicator I on PC k, *U_ik_​* is the indicator loading, and *e_k_*​ is the PC eigenvalue. Indicators within 10% of the highest *N_ik_* were identified as highly weighted (Yu et al., 2023). If multiple high-weight indicators were not significantly correlated (Pearson correlation), all were retained in the MDS; if correlated, only the indicator with the highest norm was retained (Li et al., 2013; Jin et al., 2021). MDS indicators were scored using a nonlinear scoring function (SNL), preferred for its ability to reflect soil functional responses (Raiesi and Beheshti, 2022):


$$S_{NL}=\frac{a}{1+{\left(\frac{x}{x_{m}}\right)}^{b}}$$

Where *a* = 1 is the maximum score, *x* is the observed value, *x_m_​* is the mean value, and *b* = −2.5 or 2.5 for "more is better" or "less is better" indicators, respectively (Zhang et al., 2011; Yu et al., 2018). Most indicators were scored as "more is better," except for bulk density (BD), which was scored as "less is better."

The overall Soil Quality Index (SQI) was calculated using a weighted additive model:


$$SQI=\sum_{i=1}^{n}W_{i}\cdot S_{i}$$

Where *W_i_​* is the weight of indicator *i* based on its contribution to PC variance, and *S_i_​* is its SNL score. Indicator weights were calculated as:


$$W_{i}=\frac{C_{i}}{\sum_{1}^{n}C_{i}}$$

With *C_i_* representing the common factor variance of indicator *i* (Rahmanipour et al., 2014). The SQI was normalized to a 0–1 scale and categorized into five classes: very high (>0.55), high (0.46–0.55), medium (0.37–0.46), low (0.28–0.37), and very low (<0.28), based on national standards for soil fertility classification (ISMAPRC, 1996).

To evaluate the sensitivity of SQI to different hybridization models, a sensitivity index (SI) was calculated:


$$SI=\frac{SQI_{max}}{SQI_{min}}$$

Higher *SI* values indicate greater responsiveness of soil quality to hybridization pattern (Mamehpour et al., 2021).

### 2.5 Statistical analysis

Data were processed and analyzed using Microsoft Excel 2010 and SPSS 25.0 (Statistical Graphics Corp., Princeton, USA). The Shapiro–Wilk test was used to assess data normality, and Levene’s test was applied to evaluate the homogeneity of variances. When assumptions of normality or homoscedasticity were violated, data were log-transformed prior to analysis.

One-way analysis of variance (ANOVA) was conducted to examine differences in diameter at breast height (DBH), tree height (TH), percentage of broadleaf species (PBS), coefficient of variation of DBH (CV_D_), Gini coefficient of DBH (Gini_D_), forest biomass (FB), net primary productivity (NPP), and soil properties across different mixed planting patterns. Duncan’s multiple range test was used for *post hoc* comparisons. All results are presented as mean ± standard error (SE), based on three replicates (n = 3). Pearson correlation and Mantel tests were employed to evaluate the relationships between soil properties, soil quality, stand structure, and productivity. Partial least squares regression (PLSR) was used to assess the relationship between the soil quality index (SQI), stand structural attributes, and the proportion of broadleaf species. Random forest analysis was conducted to determine the relative importance of soil properties in explaining variation in stand productivity and the proportion of broadleaf species. A partial least squares path modeling (PLS-PM) analysis was performed using the "plspm" package in R to investigate the key drivers influencing net primary productivity.

## 3 Results

### 3.1 Structural diversity and forest productivity

**Figure 2:**
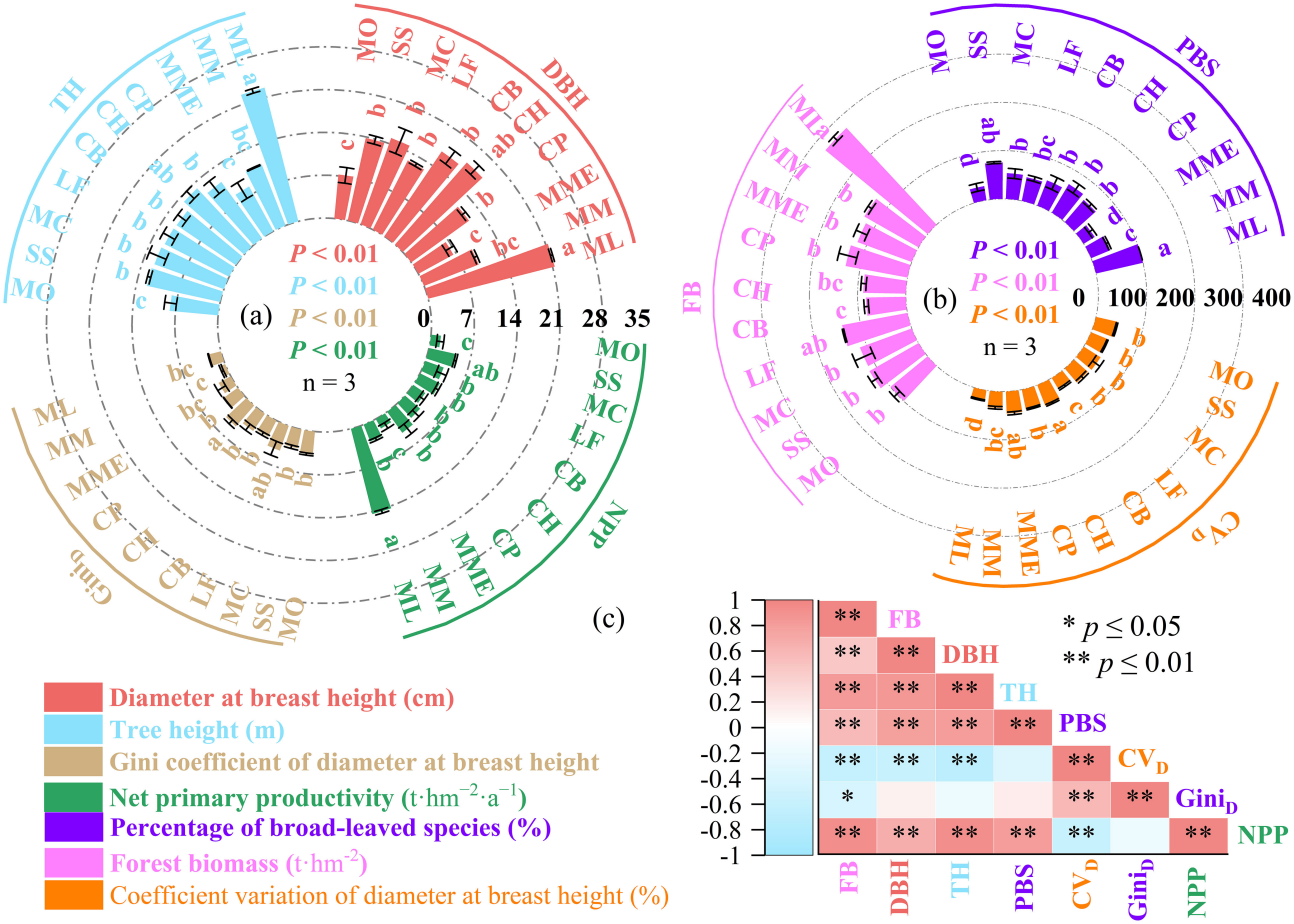
Effects of ten mixed planting patterns (ML, Fir–Mytilaria laosensis mixed; MM, Fir–Michelia macclurei mixed; MME, Fir–Michelia maudiae mixed; CP, Fir–Cinnamomum porrectum mixed; CH, Fir–Castanopsis hystrix mixed; CB, Fir–Cinnamomum burmanni mixed; LF, Fir–Liquidambar formosana mixed; MC, Fir–Michelia chapensis mixed; SS, Fir–Schima superba mixed; MO, Fir–Michelia odora mixed.) on forest structural diversity and productivity. **(a)** Net primary productivity (NPP), diameter at breast height (DBH), tree height (TH), and the Gini coefficient of DBH (Gini_D_); **(b)** percentage of broadleaf species (PBS), coefficient variation of DBH (CV_D_), and forest biomass (FB); **(c)** Pearson correlation matrix depicting the relationships among all stand structural and productivity-related variables. * indicates that a correlation is significant (P < 0.05); ** indicates that a correlation is extremely significant (P < 0.01).

The values of DBH (6.52–21.34 cm), TH (7.96–22.55 m), Gini_D_ (0.83–4.54), NPP (1.62–14.38 t·hm^-^²·a^-^¹), PBS (27.13%–100%), FB (82.78–274.18 t·hm^-^²), and CV_D_ (21.22%–46.17%) varied significantly across different mixed planting patterns (*p*< 0.05; **Figures 2a, b**). Notably, the highest values of FB, DBH, TH, and PBS were all observed in the ML planting pattern, while the highest CV_D_ and Gini_D_ values were recorded in CH. The lowest values of DBH and PBS were found in MME, whereas the lowest FB, TH, CV_D_, and Gini_D_ values were observed in CB, MO, ML, and MM, respectively. The NPP of the ML planting pattern was significantly higher than that of MO (**Figures 2a, b**).

Correlation analysis revealed that FB was significantly positively correlated with DBH, TH, and PBS. In addition, DBH showed significant positive correlations with both TH and PBS, and PBS was also positively correlated with TH. A strong positive correlation was found between Gini_D_ and CV_D_. In contrast, CV_D_ was significantly negatively correlated with DBH, TH, and FB, and Gini_D_ showed a significant negative correlation with FB. NPP was significantly correlated with all variables except Gini_D_. Specifically, NPP showed significant positive correlations with DBH, TH, PBS, and FB, and a significant negative correlation with CV_D_ (**Figure 2c**).

### 3.2 Soil properties of different mixed planting patterns

The results of the one-way ANOVA indicated that most physical (SM), chemical (TN, TP, SOC, NH_4_^+^, NO_3_^-^, AP), microbial (MBC, MBN, MBP), and enzyme activity (POD, LAP, BX, ALP, URE, CBH, NAG, ACP, and BG) characteristics, with the exception of BD, exhibited significant differences among the 10 mixed planting patterns (**Table 2**).

**Table 2:** The soil properties of ten 10 mixed planting patterns (mean ± stand and deviation, n=3).

Forest type	Physical properties		Chemical properties					
	SM (%)	BD (g·cm^-3^)	TN (g·kg^-1^)	TP (g·kg^-1^)	SOC (g·kg^-1^)	NH_4_^+^ (ug·g^-1^)	NO_3_^-^ (ug·g^-1^)	AP (ug·g^-1^)
ML	33.08 ± 3.55^ab^	1.08 ± 0.07	1.59 ± 0.12^ab^	0.35 ± 0.02^b^	25.94 ± 2.37^ab^	2.56 ± 0.27^ab^	0.29 ± 0.02^b^	1.15 ± 0.03^a^
MM	29.50 ± 2.25^ab^	1.36 ± 0.09	1.93 ± 0.18^a^	0.36 ± 0.01^b^	28.54 ± 3.04^a^	2.61 ± 0.27^ab^	0.36 ± 0.01^b^	0.53 ± 0.02^ab^
MME	35.11 ± 1.82^a^	1.20 ± 0.03	1.66 ± 0.08^ab^	0.40 ± 0.01^ab^	23.39 ± 3.90^ab^	5.99 ± 0.60^ab^	1.24 ± 0.03^a^	0.76 ± 0.08^ab^
CP	25.72 ± 1.17^c^	1.33 ± 0.06	1.68 ± 0.12^ab^	0.30 ± 0.02^c^	25.76 ± 1.98^ab^	3.07 ± 0.19^ab^	0.70 ± 0.07^ab^	0.43 ± 0.04^ab^
CH	25.32 ± 0.14^c^	1.25 ± 0.13	1.32 ± 0.08^b^	0.35 ± 0.01^b^	17.68 ± 1.47^b^	3.75 ± 0.18^ab^	1.17 ± 0.07^a^	0.62 ± 0.06^ab^
CB	27.42 ± 0.94^b^	1.36 ± 0.09	1.02 ± 0.10^c^	0.49 ± 0.04^a^	12.85 ± 1.11^c^	5.61 ± 0.48^ab^	0.17 ± 0.07^c^	0.45 ± 0.04^ab^
LF	30.65 ± 1.67^ab^	1.23 ± 0.10	1.79 ± 0.07^ab^	0.40 ± 0.02^ab^	23.54 ± 0.14^ab^	4.25 ± 0.26^ab^	1.22 ± 0.12^a^	0.16 ± 0.01^c^
MC	31.23 ± 2.84^ab^	1.42 ± 0.16	1.48 ± 0.13^ab^	0.33 ± 0.04^b^	19.80 ± 1.05^ab^	0.53 ± 0.05^b^	0.30 ± 0.02^b^	0.34 ± 0.02^b^
SS	27.52 ± 1.84^b^	1.24 ± 0.07	1.84 ± 0.13^ab^	0.46 ± 0.04^ab^	27.09 ± 3.05^ab^	1.33 ± 0.04^ab^	0.37 ± 0.02^b^	0.68 ± 0.03^ab^
MO	32.51 ± 1.78^ab^	1.24 ± 0.07	1.88 ± 0.15^ab^	0.33 ± 0.03^b^	28.46 ± 1.84^a^	10.18 ± 0.98^a^	0.95 ± 0.07^ab^	0.56 ± 0.06^ab^
Forest type	Microbiological properties			Enzyme activity properties				
	MBC (mg·kg^-1^)	MBN (mg·kg^-1^)	MBP (ug·g^-1^)	POD (μmol·d^-1^·g^-1^)	LAP (μmol·d^-1^·g^-1^)	BX (μmol·d^-1^·g^-1^)	ALP (μmol·d^-1^·g^-1^)	URE (μmol·d^-1^·g^-1^)
ML	868.11 ± 93.56^b^	56.74 ± 5.96^ab^	2.05 ± 0.13^ab^	71.71 ± 1.61^a^	6.82 ± 0.61^b^	6.21 ± 0.50^c^	12.45 ± 1.32^b^	304.95 ± 30.79^a^
MM	996.36 ± 20.95^ab^	49.25 ± 3.95^ab^	1.91 ± 0.16^b^	34.54 ± 3.44^bc^	7.77 ± 0.64^b^	7.72 ± 0.56^b^	11.69 ± 0.48^b^	204.11 ± 13.62^b^
MME	1609.83 ± 146.93^a^	73.24 ± 5.87^ab^	3.70 ± 0.31^ab^	49.30 ± 3.71^ab^	13.66 ± 0.91^a^	8.08 ± 0.30^b^	15.99 ± 0.75^ab^	260.78 ± 27.86^ab^
CP	1130.53 ± 114.38^ab^	41.45 ± 0.44^ab^	3.90 ± 0.04^a^	41.34 ± 1.48^b^	13.71 ± 0.54^a^	8.41 ± 0.80^ab^	11.39 ± 0.75^b^	191.68 ± 15.32^b^
CH	827.26 ± 49.62^c^	44.37 ± 3.23^ab^	1.87 ± 0.10^b^	28.16 ± 0.56^c^	12.67 ± 1.15^a^	8.20 ± 0.34^b^	6.75 ± 0.34^c^	221.82 ± 21.34^b^
CB	939.05 ± 77.53^ab^	39.15 ± 2.82^b^	1.82 ± 0.80^b^	27.83 ± 3.13^c^	8.37 ± 0.67^b^	7.46 ± 0.53^b^	8.11 ± 0.66^c^	313.14 ± 26.56^a^
LF	1103.98 ± 74.90^ab^	85.37 ± 4.29^a^	1.85 ± 0.20^b^	26.44 ± 2.06^c^	13.54 ± 0.67^a^	8.92 ± 0.25^ab^	14.24 ± 0.86^ab^	224.48 ± 21.30.^b^
MC	979.58 ± 78.80_ab_	53.14 ± 2.30^ab^	1.83 ± 0.09^b^	37.36 ± 3.36^b^	13.24 ± 1.43^a^	7.07 ± 0.51^b^	10.90 ± 0.88^b^	272.31 ± 28.54^ab^
SS	1220.35 ± 79.04^ab^	69.44 ± 3.62^ab^	1.54 ± 0.17^b^	28.05 ± 3.14^c^	14.29 ± 1.34^a^	8.88 ± 0.87^ab^	5.89 ± 0.60^c^	164.74 ± 12.41^c^
MO	1413.23 ± 55.00^ab^	96.27 ± 3.05^a^	2.84 ± 0.07^ab^	37.14 ± 2.39^b^	8.15 ± 0.83^b^	10.54 ± 1.21^a^	18.43 ± 1.57^a^	179.35 ± 7.81^c^
Forest type	Enzyme activity properties							
	CBH (μmol·d^-1^·g^-1^)	NAG (μmol·d^-1^·g^-1^)	ACP (μmol·d^-1^·g^-1^)	BG (μmol·d^-1^·g^-1^)				
ML	38.74 ± 1.65^a^	39.96 ± 4.50^b^	14.66 ± 0.40^c^	45.77 ± 3.45^bc^	See **Appendix II** for abbreviations, different lowercase letters indicate statistically significant differences at *P* < 0.05 level.			
MM	18.66 ± 1.47^b^	44.34 ± 2.41^b^	15.68 ± 0.39^b^	25.64 ± 1.38^c^				
MME	25.08 ± 1.90^ab^	38.34 ± 1.46^b^	15.89 ± 0.34^b^	56.22 ± 3.37^b^				
CP	17.44 ± 0.57^b^	25.79 ± 1.74^bc^	19.52 ± 1.61^ab^	116.82 ± 10.88^ab^				
CH	17.46 ± 0.45^b^	17.98 ± 0.81^c^	14.81 ± 0.60^c^	17.80 ± 1.99^c^				
CB	27.47 ± 2.21^ab^	59.75 ± 5.68^ab^	15.53 ± 0.19^b^	61.54 ± 3.06^b^				
LF	17.41 ± 1.15^b^	14.48 ± 0.60^c^	18.93 ± 1.60^b^	51.59 ± 4.49^b^				
MC	25.08 ± 1.88^ab^	72.97 ± 5.22^a^	24.82 ± 1.49^a^	63.89 ± 5.19^b^				
SS	18.06 ± 1.05^b^	15.50 ± 1.19^c^	17.81 ± 1.62^b^	240.99 ± 16.00^a^				
MO	15.13 ± 0.14^b^	13.88 ± 0.78^c^	15.05 ± 0.75^c^	108.48 ± 6.45^ab^				

### 3.3 Soil quality assessment

Principal component analysis (PCA) identified six principal components with eigenvalues greater than 1, which were selected for constructing the Minimum Data Set (MDS). The eigenvalues of the components were as follows: PC1 = 6.366, PC2 = 2.196, PC3 = 1.948, PC4 = 1.759, PC5 = 1.368, and PC6 = 1.101. Collectively, these six components explained 73.68% of the total variance in the dataset (**Table 3**).

**Table 3:** Load matrix communality value and norm values of assessment indicators.

Soil parameter	Principal component						Norm value	Communality value	Group
	PC1	PC2	PC3	PC4	PC5	PC6			
SM	0.598	-0.374	0.012	-0.017	-0.267	-0.083	1.640	0.575	1
BD	-0.552	0.229	0.125	0.25	0.214	0.021	1.503	0.481	1
TN	0.849	0.222	0.157	-0.029	0.051	-0.142	2.185	0.819	1
TP	0.042	-0.102	0.004	0.269	0.545	0.628	1.001	0.776	6
SOC	0.853	0.205	0.201	-0.115	-0.026	-0.126	2.201	0.839	1
MBC	0.845	0.117	-0.171	0.094	0.154	0.045	2.164	0.791	1
NH_4_^+^-	0.484	-0.178	-0.685	0.093	0.036	-0.010	1.579	0.745	3
NO_3_^–^	0.162	0.608	-0.347	-0.248	0.363	0.135	1.234	0.728	2
MBN	0.85	0.102	-0.102	0.177	0.025	0.007	2.168	0.775	1
MBP	0.733	0.222	-0.166	-0.197	0.295	0.025	1.942	0.741	1
AP	0.547	-0.344	-0.163	-0.428	0.004	0.344	1.634	0.745	1
POD	0.375	-0.245	0.385	-0.617	-0.122	0.112	1.421	0.757	4
LAP	0.572	0.228	0.165	0.461	-0.182	0.057	1.635	0.655	1
BX	0.176	-0.173	-0.483	0.672	-0.321	0.026	1.286	0.85	4
ALP	0.895	-0.007	0.009	0.079	-0.053	-0.122	2.265	0.825	1
URE	0.578	-0.363	0.459	0.164	0.145	-0.128	1.709	0.74	1
CBH	0.142	-0.653	0.112	0.104	-0.136	0.444	1.162	0.686	2
NAG	0.037	-0.349	0.485	0.362	0.556	-0.184	1.194	0.833	5
ACP	0.255	0.473	0.465	0.225	-0.134	0.127	1.207	0.59	2
BG	0.023	0.514	0.317	0.072	-0.430	0.479	1.138	0.784	2
Principal component eigenvalue	6.366	2.196	1.948	1.759	1.368	1.101			
Contribution rate (%)	31.828	10.980	9.739	8.795	6.839	5.504			
Accumulating contribution rate (%)	31.828	42.808	52.547	61.341	68.181	73.684			

Based on PCA results, all 20 soil indicators were grouped by principal component as follows:

- Group 1: SM, BD, TN, SOC, MBC, MBN, MBP, AP, LAP, ALP, URE
- Group 2: NO_3_^-^, CBH, ACP, BG
- Group 3: NH_4_^+^
- Group 4: POD, BX
- Group 5: NAG
- Group 6: TP

From each group, indicators with high loadings (within 10% of the group’s highest norm value) were initially retained. These included:

- Group 1: ALP, SOC, TN, MBN, MBC
- Group 2: NO_3_^-^, CBH, ACP, BG
- Groups 3–6: NH_4_^+^, POD, BX, NAG, TP (each the sole indicator in its group)

**Figure 3:**
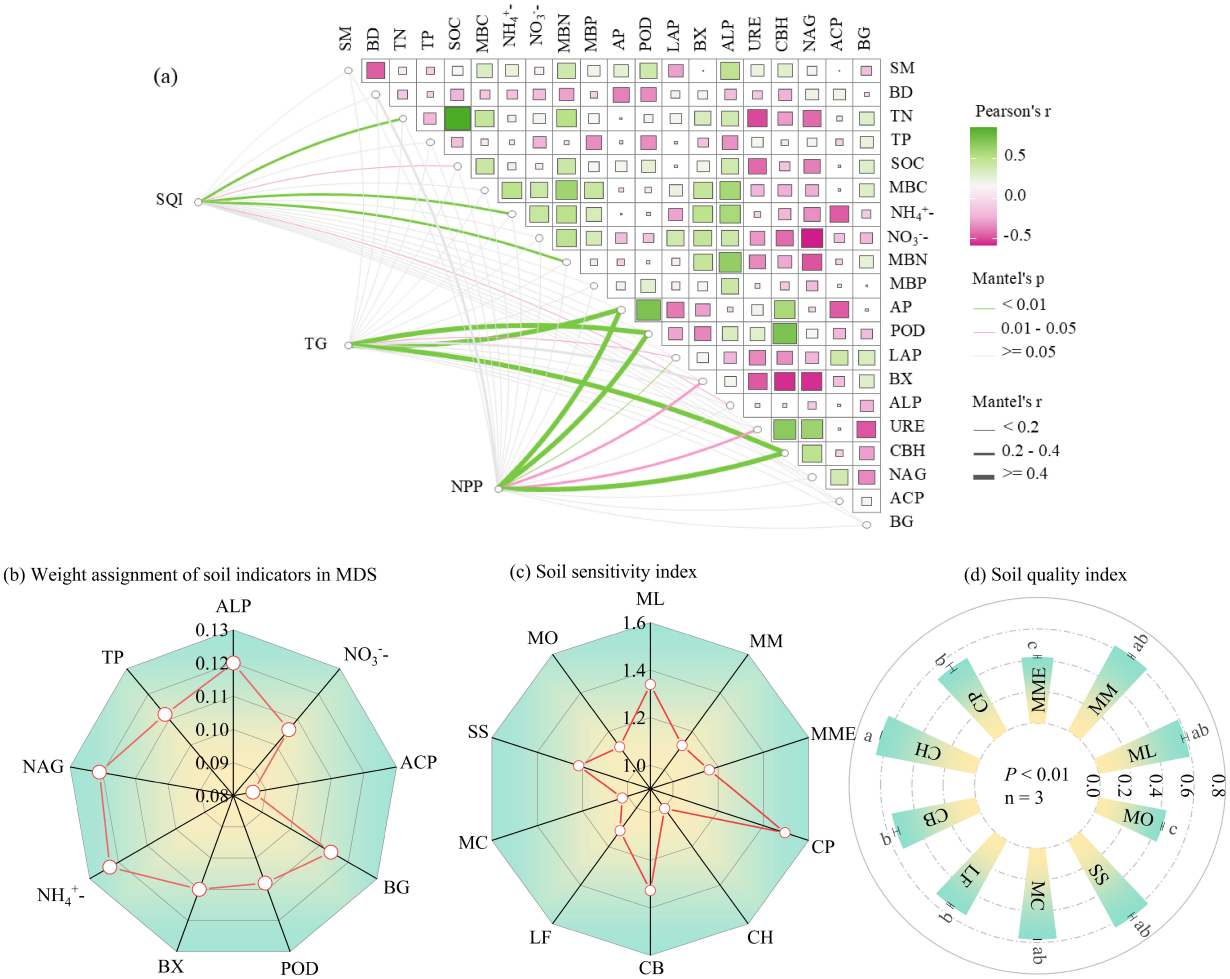
The Pearson correlation between soil indicators and the Mantel test concerning the soil quality index, tree growth metrics (DBH, TH, CV_D_, and Gini_D_), and net primary productivity (NPP) in relation to soil properties **(a)**, weight assignment soil indicators in MDS **(b)**, soil sensitivity index **(c)** and Soil quality index **(d)**. The figure **(d)** value reported as mean ± standard deviation (n = 3). Significant differences at *p* < 0.05 are indicated by different lowercase letters. Classification of soil quality, “very high” (SQI>0.55), “high” (0.46-0.55), “medium” (0.37-0.46), “low” (0.28-0.37) and “very low” (SQI<0.28) (Guo et al., 2017).

Correlation analysis (**Figure 3a**) was then used to further refine indicator selection. In Group 1, ALP showed significant correlations with SOC (r = 0.40*), TN (r = 0.37*), MBC (r = 0.59**), and MBN (r = 0.62**), and was therefore selected as the representative indicator due to its highest norm value. In Group 2, NO_3_^-^ and CBH were significantly negatively correlated (r = –0.38*), so NO_3_^-^, ACP, and BG were retained. In Group 4, POD and BX were not significantly correlated and were both included. In Groups 3, 5, and 6, NH_4_^+^, NAG, and TP were each retained by default as the only high-loading indicators in their respective groups.

Ultimately, the final MDS consisted of nine indicators: ALP, NO_3_^-^, ACP, BG, POD, BX, NH_4_^+^, NAG, and TP. The assigned weights for these indicators were 0.120, 0.106, 0.086, 0.114, 0.108, 0.110, 0.123, 0.121, and 0.112, respectively (**Figure 3b**).

The soil sensitivity indices (SI) for the ten mixed planting patterns ranged from 1.002 to 1.496, with CP exhibiting the highest sensitivity and CH the lowest. Other planting patterns with relatively high SI values included ML (1.340), CB (1.327), and SS (1.217) (**Figure 3c**). Significant differences in Soil Quality Index (SQI) were observed among the ten planting patterns (*p*< 0.05), with values ranging from 0.42 to 0.65. CH had a significantly higher SQI than both MME and MO (**Figure 3d**). According to the SQI classification (Guo et al., 2017), all ten mixed planting patterns were rated at or above the medium level: ML, MM, CH, MC, and SS were classified as very high; CP, CB, and LF as high; and MME and MO as medium.

**Figure 4:**
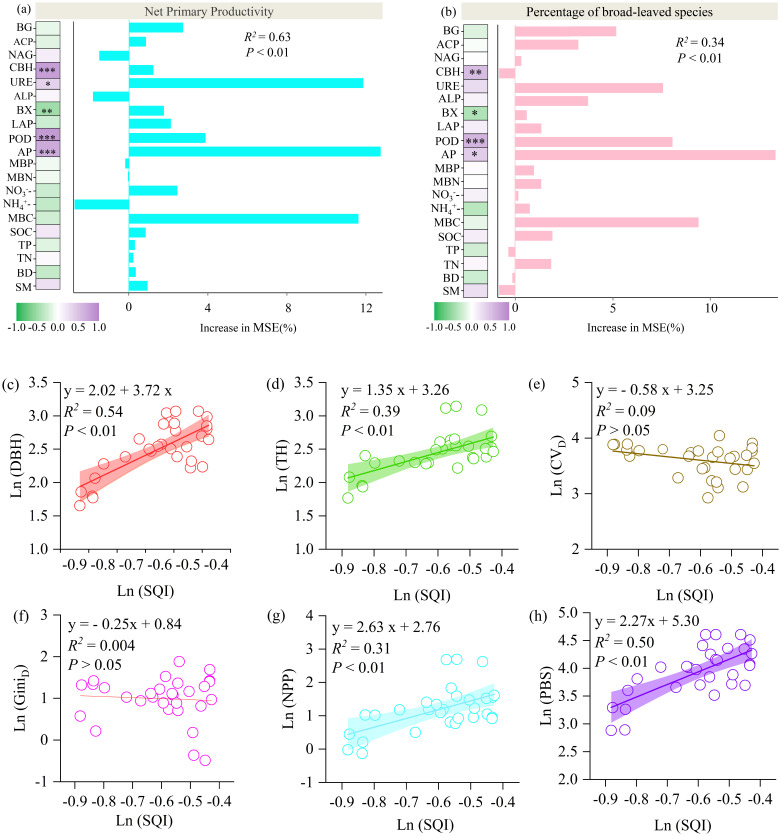
Relative importance of soil variables in predicting variations in **(a)** net primary productivity and **(b)** the percentage of broad-leafed species in mixed plantations. The least-squares regression analysis includes SQI and the following variables: **(c)** DBH, **(d)** TH, **(e)** CV_D_; **(f)** Gini_D_, **(g)** NPP, **(h)** PBS. All data were logarithmically transformed prior to the fitting analysis. Statistically significant relationships (P < 0.05) are indicated, with the 95% confidence intervals represented by the shaded areas. Asterisks denote significant correlations at the * P < 0.05, ** P < 0.01, and *** P < 0.001 levels, respectively.

Among the soil properties, urease activity (URE), available phosphorus (AP), and microbial biomass carbon (MBC) had the strongest influence on stand productivity. Additionally, URE, AP, MBC, and Peroxidase (POD) were significant predictors of the proportion of broadleaf species in mixed stands (**Figures 4a, b**).

Stand structural diversity, as measured by the coefficient of variation in DBH (CV_D_) and the Gini coefficient (Gini_D_), showed no significant correlation with SQI (**Figures 4e, f**). In contrast, tree height (TH), DBH, net primary productivity (NPP), and the proportion of broadleaf species increased significantly with higher SQI values (*p*< 0.01; **Figures 4c, d, g, h**).

**Figure 5:**
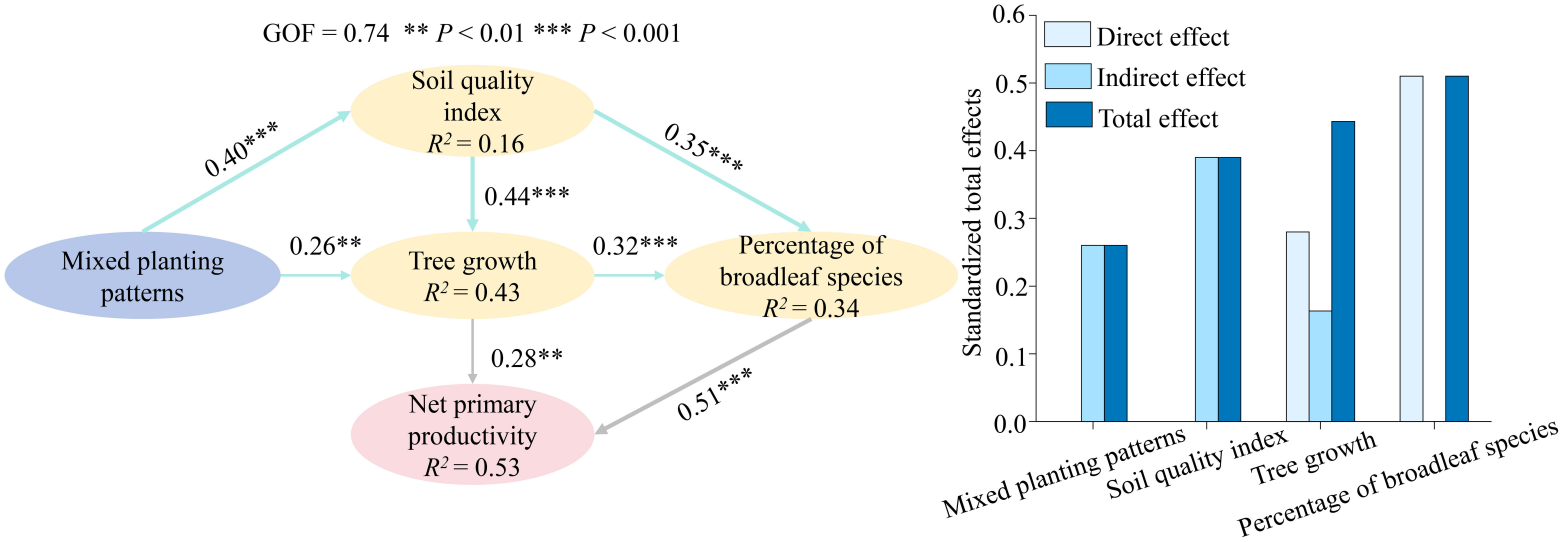
Partial least squares path modeling (PLS-PM) describing the direct and indirect effects of mixed planting patterns on net primary productivity. The model demonstrates a good goodness of fit (GOF = 0.74). Path coefficients are displayed alongside the arrows, with significance levels indicated as ** (*P* < 0.01) and *** (*P* < 0.001). The *R²* values represent the proportion of variance explained for each endogenous variable. Gray single-headed arrows denote direct effects on net primary productivity, while blue arrows represent the direct effects of mixed planting patterns on mediating variables (soil quality index, tree growth [DBH, TH, CV_D_, Gini_D_], percentage of broadleaf species), which subsequently exert indirect effects on net primary productivity.

The PLS-PM model confirms that mixed planting patterns do not directly enhance net primary productivity to a great extent but rather function by initiating a key ecological succession process. Specifically, these patterns improve the soil quality and promote tree growth, which in turn drives the stand composition toward a higher percentage of broadleaf species. Ultimately, this increased percentage of broadleaf species acts as the most powerful direct driver, significantly boosting the forest's net primary productivity. This finding underscores the pivotal role of species composition, particularly the proportion of broadleaf species, in shaping the productivity of mixed forests (**Figure 5**).

## 4 Discussion

### 4.1 Growth and soil characteristics of forest stands

Previous studies have shown that mixed-species plantations can enhance ecosystem productivity and improve ecosystem functions and services relative to the original single-species plantation (Guo et al., 2023). Near-naturalization of coniferous forests—through structural transformation of homogeneous monocultures into mixed stands—has been proposed as a strategy to achieve this (Hou et al., 2021; Guo et al., 2022). In this study, different combinations of fir and broadleaf species resulted in substantial differences in stand growth (DBH and TH), structural diversity (CV_D_ and Gini_D_), and total biomass (**Figure 2**). These differences likely stem from interspecific variation in shade tolerance and growth rates, which leads to vertical stratification of the forest canopy (Thurm and Pretzsch, 2016).

Interestingly, stand structural diversity in this study was negatively correlated with both stand growth and biomass (**Figure 2**). This contrasts with findings from other studies reporting positive relationships between structural diversity and aboveground biomass, including in temperate mixed forests of northeastern China (Yuan et al., 2018), subtropical secondary forests (Ali et al., 2016), and large-scale tropical forests (Ali et al., 2019). For example, Zhang and Chen (2015) reported that tree diameter diversity was positively correlated with aboveground biomass, and Dănescu et al. (2016) highlighted the direct contribution of structural diversity to productivity. The discrepancy between our results and previous findings may be attributed to differences in community composition, species-specific traits, and environmental conditions (Tetemke et al., 2021; Ullah et al., 2021). It has also been proposed that negative relationships between structural diversity and productivity can emerge under competitive exclusion and selection effects, particularly in less-disturbed or high-productivity environments (Paquette and Messier, 2011).

Most soil physical, chemical, microbial, and enzymatic indicators—excluding bulk density (BD)—exhibited significant variation among the mixed planting types (**Table 2**), but no single forest type consistently exhibited the highest soil values. This suggests that the effects of species mixing on soil properties are not uniform. One potential explanation is that some of the broadleaf evergreens introduced have relatively low litter inputs, influencing soil structure and function (Zhou et al., 2020). Past studies have similarly shown that shifts in soil physicochemical characteristics depend heavily on the vegetation types used to restore degraded lands (Hou et al., 2019), and species-specific effects in mixed stands can further mediate these changes (Stewart et al., 2011). Overall, this underscores the importance of rational species selection in reforestation strategies to enhance soil improvement in degraded fir plantations.

### 4.2 Soil quality index and forest succession

Monitoring and evaluating soil quality is a critical component of sustainable forest management (Huang et al., 2018). The Soil Quality Index (SQI), developed from a multivariate indicator set, provides a quantitative framework to evaluate the impact of management practices on soil health (Chen et al., 2021; He et al., 2022). In this study, an MDS of nine indicators—including three physicochemical properties (TP, NO_3_^-^, NH_4_^+^) and six enzyme activity metrics (ALP, ACP, BG, POD, BX, NAG)—was selected to simplify and strengthen the SQI assessment. The inclusion of enzyme indicators involved in C, N, and P cycling reflects their value in capturing biologically mediated changes in soil function. These findings refine and support previous calls to include enzyme activities in soil quality assessments for cedar forests.

The observed SQI values (0.42–0.65) were lower than those previously reported in other fir-broadleaf plantations (0.66–0.85; Huang et al., 2018), likely due to differences in climate, stand age, or species composition (Shao et al., 2020; Cao et al., 2023). Among the mixed planting patterns, CH, ML, MM, MC, and SS exhibited significantly higher SQI values (**Figure 3d**). Notably, the ML, MC, and SS patterns also had high stand biomass (**Figure 2a**), suggesting that these combinations not only enhance soil quality but also promote productivity. Although CH had lower biomass, it featured the highest SQI and exhibited large DBH and tree height values (**Figures 2b, c**), likely due to low stand density and dominance by larger individuals (**Table 1**). This suggests that CH may be a suitable planting model for cultivating large-diameter timber in degraded fir plantations. In contrast, the MM pattern exhibited higher soil quality but relatively low DBH and tree height (**Figure 2**), making it less suitable for timber production despite good soil performance. These differences highlight the importance of selecting mixed-species strategies tailored to specific management goals—whether soil restoration or timber yield.

Forest structural attributes, including variation in tree size (DBH and height), are known to influence species diversity, regeneration dynamics, and forest function (Clark, 2010; Yuan et al., 2018; Hui et al., 2019; Põldveer et al., 2021). In this study, the proportion of broadleaf species differed significantly across mixed stands (**Figure 2d**), likely due to species-specific requirements for light and water. High structural diversity, as seen in CH and MC, can enhance ecological niche complementarity through improved light utilization. Conversely, lower structural complexity, such as in ML, may reduce resource-use efficiency (Ali et al., 2016).

The transition from fir- to broadleaf-dominant stands was closely tied to both tree size and soil quality. Improved soil quality significantly promoted DBH and height (**Figure 4**), which in turn increased the percentage of broadleaf trees in mixed stands. These findings are consistent with the idea that nutrient-rich soils facilitate growth and canopy development (Poorter et al., 2017), leading to changes in vertical structure and light interception (Onoda et al., 2014).

Forest structure also mediates competition and regeneration. As tree size variation increases, dominant individuals face reduced competition while suppressed individuals experience intensified resource limitation (Ali and Yan, 2017; Zhang et al., 2017). Size-asymmetric competition—particularly for light—is a major mechanism driving tree mortality and skewed size distributions (Bourdier et al., 2016; Soares et al., 2016). In our study, although the initial planting densities were uniform, stand density diverged considerably over time (**Table 1**), highlighting the dynamic nature of post-establishment competition. Fir, being shade-intolerant, initially grows faster than broadleaf species, but is eventually outcompeted under closed-canopy conditions as broadleaf trees capture more light and water due to their larger canopies (Zhang et al., 2020).

As upper-canopy trees escape light limitation, suppressed fir individuals face increasingly severe shading. This can lead to carbon starvation, where photosynthetic gains are insufficient to cover respiratory costs (Luo and Chen, 2011; Zhang et al., 2020). Larger Gini coefficients reflect increased size inequality and intensified asymmetric competition, which can accelerate mortality of smaller individuals. This is supported by our observation that ML, which had larger average DBH and tree height, also exhibited lower Gini coefficients—suggesting a more uniform, less competitive size structure.

One limitation of this study is the absence of baseline soil data from the plantation establishment period (circa 2003). This precludes us from definitively ruling out the potential influence of initial soil heterogeneity on the observed community trajectories. Moreover, the strong correlations between current soil properties (e.g., URE, AP, MBC) and stand attributes (DBH, TH, PBS) likely reflect bidirectional plant-soil feedbacks rather than a unidirectional soil effect. For instance, introducing broadleaf species can initiate positive feedback cycles by enhancing nutrient availability and microbial activity via litter and root exudates, which in turn promotes their growth and dominance (Zhou et al., 2020; Guo et al., 2023). Nevertheless, significant differences in SQI and stand structure emerged after two decades from a common starting point—a uniformly degraded fir plantation. Coupled with the identification of key, dynamic soil drivers (e.g., URE, MBC) that are responsive to vegetation change, this provides compelling evidence that soil quality enhancement is a critical mechanism driving successional transition. Thus, our findings reveal a strong, functionally important plant-soil feedback loop that governs succession in these mixed plantations, rather than a simple unidirectional pathway.

## 5 Conclusions

This study evaluated soil properties, stand growth, structural diversity, biomass, and the proportion of broadleaf species across various fir–broadleaf mixing patterns. The results demonstrated that differences in soil properties were primarily influenced by species composition. Among the planting patterns, ML, CH, MC, and SS significantly improved both soil quality and stand biomass. Enhanced soil quality played a key role in promoting tree growth, thereby accelerating the near-natural successional transition from fir-dominated stands to broadleaf forests. For the conversion of degraded Chinese fir plantations, the species MC, ML, and CH are proposed as suitable candidates to enhance ecosystem productivity and soil quality. These findings underscore the importance of rational species selection in rehabilitating degraded cedar plantations and highlight soil quality as a critical driver of successional dynamics in mixed stands. This work offers new insights into the management and restoration of degraded fir plantations and presents a foundation for the development of ecologically informed hybridization strategies.

## Data Availability

The original contributions presented in the study are included in the article/**Supplementary Material**. Further inquiries can be directed to the corresponding author.
