# Supplementary material for: Soil quality enhancement drives tree growth and broadleaf dominance in fir-broadleaf mixed plantations
Source: Front Plant Sci. 2025 Nov 19;16:1705626. doi: 10.3389/fpls.2025.1705626 (PMC12672489; doi:10.3389/fpls.2025.1705626)
Supplement: Supplementary file 1 [file Table1.docx]

Appendix Ⅰ

The analysis of soil properties

| Abbreviation | Full name | Method |
| --- | --- | --- |
| SM | Soil moisture content | Samples were oven-dried until attained a constant weight |
| BD | Bulk density | Ring-knife |
| TN | Total nitrogen | Kjel­dahl |
| TP | Total phosphorus | Molybdenum-antimony |
| SOC | Soil organic carbon | Potassium dichromate oxidation |
| MBC | Microbial biomass carbon | Chloroform fumigation-extraction |
| MBN | Microbial biomass nitrogen |  |
| MBP | Microbial biomass phosphorus |  |
| NH_4_^+^- | Ammonium nitrogen | 2 mol L^−1^ KCl indophenol blue colorimetric |
| NO_3_^-^- | Nitrate nitrogen |  |
| AP | Available Phosphorus | Molybdenum blue colorimetry |
| LAP | Leucine aminopeptidase | Fluorometric microplate enzyme assay |
| NAG | N-acetyl-β-D-glucosaminidase |  |
| CBH | Cellobiohydrolase |  |
| BG | β-1,4-glucosidase |  |
| ACP | Acid phosphatase |  |
| ALP | Alkaline phosphatase |  |
| POD | Peroxidase |  |
| BX | Xylosidase |  |
| URE | Urease |  |

Appendix II

Biomass estimation models of tree species

| Species | Organs | Model parameters | | |
| --- | --- | --- | --- | --- |
|  |  | *a* | *b* | *R^2^* |
| *Fir(Schima superba)* | Trunk | 0.0207 | 0.9935 | 0.93 |
|  | Branch | 0.0503 | 0.7183 | 0.96 |
|  | Leaf | 0.0095 | 0.7609 | 0.96 |
|  | Root | 0.0225 | 0.8178 | 0.93 |
| *Liquidambar formosana* | Trunk | 0.0465 | 0.9579 | 0.96 |
|  | Branch | 0.0099 | 0.9377 | 0.95 |
|  | Leaf | 0.0006 | 1.1187 | 0.83 |
|  | Root | 0.0164 | 0.9547 | 0.96 |
| *Cunninghamia lanceolata* | Trunk | 0.0474 | 0.8247 | 0.99 |
|  | Branch | 0.0176 | 0.7416 | 0.98 |
|  | Leaf | 0.0671 | 0.5189 | 0.94 |
|  | Root | 0.3303 | 0.4088 | 0.85 |
| Other hardwood tree species（*Michelia maudiae*、*Cinnamomum porrectum*、*Cinnamomum burmanni*、*Michelia chapensis*、*Michelia odora*、*Michelia macclurei*、*Mytilaria laosensis*） | Trunk | 0.0545 | 0.8630 | 0.89 |
|  | Branch | 0.0155 | 0.8737 | 0.89 |
|  | Leaf | 0.0145 | 0.7444 | 0.89 |
|  | Root | 0.3007 | 0.827 | 0.89 |
| *Castanopsis hystrix* | Trunk | 0.01344 | 1.0129 | 0.99 |
|  | Branch | 0.00110 | 1.1206 | 0.96 |
|  | Leaf | 0.01460 | 0.6881 | 0.96 |
|  | Root | 0.00284 | 1.0460 | 0.99 |
